# Progress in Diseases of the Heart

**Published:** 1904-02-27

**Authors:** 


### Progress in Diseases of the Heart.

(*Concluded from page* 357.)

**Diastolic Basic Murmurs without Lesions of the Aortic or Pulmonary Valves.**—Cabot and Locke[5] as the result of post-mortem examinations come to the following conclusions. Diastolic murmurs without organic valve lesions are not uncommon in connection with dilatation of the aorta, localised or diffused. When the pleura and pericardium are adherent, owing to tuberculosis or other causes, diastolic murmurs are occasionally audible in the præcordia; they are notably affected by respiration and position, and are probably due, in most cases, to suction or pulsion exerted by the heart upon portions of the lung adherent to the pericardium (cardio-respiratory murmurs). In cases of intense anæmia one occasionally hears diastolic murmurs not to be explained by permanent dilatation of the aortic ring nor as cardio-respiratory murmurs, and not due to a diastolic accentuation of a venous hum. The cause of these murmurs is obscure.

**A Case Simulating Pulmonary Stenosis** has been published by Achesson.[6] A man, aged 28, was admitted to hospital, very weak and delirious. The apex beat was in the fifth left space, 1½ inch outside the nipple line, and the præcordial dulness passed upwards into a dull area occupying the inner part of the upper two left spaces and involving the manubrium sterni. In the second left space, ½-inch from the border of the sternum, systolic pulsation was visible, and a long thrill could be felt. The thrill was found to correspond with a long loud purring systolic murmur leading up to a loud second sound. There was also albuminuria. At the post-mortem examination, the kidneys were found to be affected with chronic parenchymatous nephritis, and both ventricles of the heart were hypertrophied. Otherwise the heart was normal, the valves were intact and competent, and the pulmonary artery was normal. In the pulmonary region, *i.e.*, the second left space, there may occasionally be heard a long rough purring murmur accompanied by a thrill. This generally indicates stenosis of the pulmonary artery; but the causes of stenosis are very difficult of diagnosis. The intravascular causes include congenital stenosis and thrombus in the vessel. The extravascular causes are pressure on the artery as by aneurysm, the ventricle of the heart and large pericardial effusions; displacement of the heart as by pleural effusion or retraction of the lung. It is consequently often a matter of great difficulty or impossible to make sure of the cause. Again, Balfour and Broadbent have shown that in some cases the murmur may be due to absence or retraction of the usually overlying edge of the left lung, so that the conus presses against the chest wall on systole. In such cases the murmur disappears on taking a deep inspiration. In the case described the cause possibly lay in the ventricular hypertrophy. There are two other conditions which simulate pulmonary stenosis; in rare cases an aortic aneurysm may bulge to the left; and in patent ductus arteriosus a loud crescendo systolic murmur followed by an accentuated second sound is heard in the second left space.

**Gelatin Treatment of Aneurysm.**—Four cases of aneurysm treated by the subcutaneous injection of gelatin solutions are recorded by Rankin,[7] who concludes that they produce a marked and speedy decrease in all the subjective symptoms presented by internal aneurysms. This relief is only explicable on the theory of a diminution in pressure effects from shrinkage in size of the aneurysmal sac; and in three out of the four cases this shrinkage, accompanied by a marked increase in the resistancy of the tumour wall, was capable of physical demonstration. The after history of the patients gave evidence that these beneficial effects were permanent.

[5] J. Hopk. Hosp. Bull, May. [6] Lancet, Sept. 26. [7] Ibid, July 11.

